# Cell assisted lipotransfer in breast augmentation and reconstruction: A systematic review of safety, efficacy, use of patient reported outcomes and study quality

**DOI:** 10.1016/j.jpra.2016.08.004

**Published:** 2016-08-24

**Authors:** Zeeshaan Arshad, Lindsey Karmen, Rajan Choudhary, James A. Smith, Olivier A. Branford, David A. Brindley, David Pettitt, Benjamin M. Davies

**Affiliations:** a School of Medicine, University of St. Andrews, St. Andrews, North Haugh, UK; b The Oxford – UCL Centre for the Advancement of Sustainable Medical Innovation (CASMI), The University of Oxford, Oxford, UK; c School of Medicine, University of Oxford, Oxford, UK; d Nuffield Department of Orthopedics, Rheumatology and Musculoskeletal Sciences, University of Oxford, Windmill Road, Oxford, UK; e The Royal Marsden NHS Foundation Trust, London, UK; f Said Business School, University of Oxford, Park End Street, Oxford, UK; g Centre for Behavioral Medicine, UCL School of Pharmacy, University College London, Brunswick Square, London, UK; h Harvard Stem Cell Institute, Divinity Avenue, Cambridge, MA, USA; i USCF-Stanford Center of Excellence in Regulatory Science and Innovation (CERSI), 4th Street, USA; j Department of Pediatrics, University of Oxford, Headley Way, UK

**Keywords:** Cell assisted lipotransfer, Breast augmentation, Breast reconstruction, Adipose derived stem cells, Stromal vascular fraction

## Abstract

**Background:**

Cell assisted lipotransfer serves as a novel technique for both breast reconstruction and breast augmentation. This systematic review assesses the efficacy, safety and use of patient reported outcome measures in studies involving cell assisted lipotransfer. We also carry out an objective assessment of study quality focussing on recruitment, follow-up and provide an up-to-date clinical trial landscaping analysis.

**Methods:**

Key electronic databases were searched according to PRISMA guidelines and pre-defined inclusion and exclusion criteria. Two independent reviewers examined the retrieved publications and performed data extraction.

**Results:**

3980 publications were identified. Following screening, 11 studies were included for full review, representing a total of 336 patients with a follow-up time ranging from six to 42 months. A degree of variation was noted in graft retention and reported satisfaction levels, although there were only three comparative studies with conflicting results. Complications occurred at a rate of 37%. Additionally, there was a paucity of objective outcomes assessments (e.g. 3D assessment modalities or validated patient reported outcome measures) in the selected studies.

**Conclusions:**

Cell assisted lipotransfer is a surgical technique that is currently employed sparingly within the plastic & reconstructive surgery community. Presently, further technical and outcome standardization is required, in addition to rigorous randomized controlled trials and supporting long-term follow-up data to better determine procedural safety and efficacy. Routine use of more objective outcome measures, particularly 3D assessments and validated patient reported outcome measures, will also help facilitate wider clinical adoption and establish procedural utility.

## Introduction

Breast augmentation was the most commonly performed cosmetic procedure in the US in 2014 with over 102,000 procedures taking place.^1^ Conventionally, implants have been utilized. However, their use is associated with a number of complications, notably capsular contraction, malposition, and anaplastic large cell lymphoma.^2^, ^3^ In a proportion of post-mastectomies, the use of implants is not possible due to the irregularity of the soft tissue defects, particularly in post-radiotherapy patients.^4^ An alternative is the employment of complex reconstructive techniques including *deep inferior epigastric perforator* (DIEP) and *latissimus dorsi* (LD) flaps, that have an inherent complication risk and longer recovery periods.^5^ Although cell assisted lipotransfer (CAL) will not replace these procedures (due to the shape and projection profiles they achieve), it has potential to serve as an adjunct for small corrections or volume increases, and may serve as a less invasive option for patients hoping to achieve subtle aesthetic enhancements. It should be noted that there is limited evidence to suggest that fat grafting without the use of adipose derived stem cell supplementation can be used for complete post-mastectomy reconstruction. This has, however, used a technique called BRAVA-assisted fat grafting were an external volume expansion device is applied to enhance graft survival. For example, Khouri 2015 conducted a level IV study on 488 women (616 breast) and concluded that BRAVA-assisted fat grafting is a minimally invasive, safe and economic alternative to other forms of breast reconstruction.^6^ The technique has also been used by the same study author to investigate the effect of large volume fat grafting after BRAVA use or implant removal with positive results.^7^ Adipose derived stem cells, fat grating and external volume expansion technology is therefore a potential area of future research, specifically with regards to whole breast and large volume fat grafting.

An option for both breast augmentation and reconstruction is autologous fat grafting. Although studies have reported a more natural breast contour, reports of fat resorption have been reported.^8^ Reported graft retention using this procedure vary from 40 to 75%, and therefore there is room for improvement in the efficacy of this procedure.^9^ It has been found that the key to fat graft retention is maximizing the surface area to volume ratio, and the vascularity of the recipient area.^10^, ^11^ Studies have suggested that adipose derived stem cells can survive the period of hypoxia post surgery that is thought to result in the necrosis of conventional fat.^11^ This provides scientific rational to using CAL in breast surgery and is why the use of this technique can be seen as a key development in the repertoire of techniques available to surgeons.^12^

**Figure 1:**
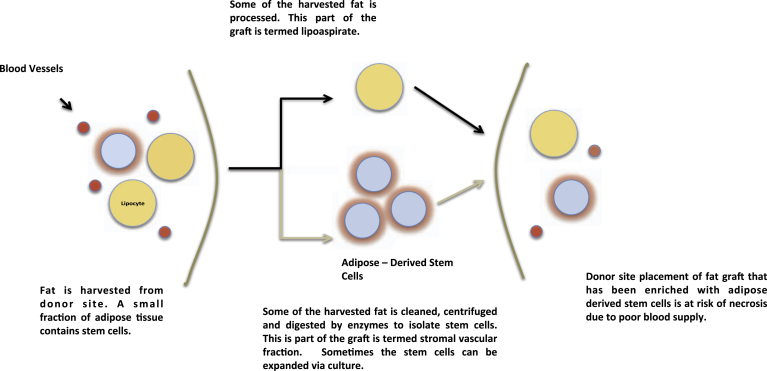
The cell assisted lipotransfer procedure. Harvested fat is separated with approximately one half being used to harvest adipose derived stem cells and the other being processed for use as a fat graft. The two constituents are then combined and placed within the recipient site. Note that the black arrows indicate the procedure for a conventional autologous fat transfer procedure.

CAL utilizes fat grafts that have been enriched with a patient's adipose derived stem cells (Figure 1 compares CAL to autologous fat transfer). Adipose derived stem cells are able to enhance both angiogenesis and adipogenesis. Translating this into the clinical setting, it is hoped that long-term graft retention and lower post-operative complication rates will result.^13^, ^14^ The abundance of adipose tissue makes the harvesting of these cells relatively easy, avoiding the need for *in-vitro* expansion.^15^ In addition, the removal of fat from aesthetically sensitive anatomical regions offers secondary patient benefits.

Several clinical studies have assessed the use of CAL in breast surgery. However, there has been no objective assessment of the evidence in this area. Limited examples of secondary research have been published examining CAL for multiple indications,^16^, ^17^ but no studies focus exclusively on breast-related procedures. In light of this and the prospective role of CAL in breast augmentation and reconstruction, this systematic review was conducted to assess the efficacy and safety of cell assisted lipotransfer in breast surgery. We also aimed to ascertain the quality of the completed studies with particular consideration to methods of recruitment, appropriate follow-up and assessment of patient reported outcomes. Through this, we aim to inform future clinical trial organizers of limitations of current studies.

## Methods

### Search strategy

A systematic review was carried out according to the PRISMA guidelines,^18^ the details of which can be found in Supplementary Material Document 1.

## Results

### Study selection

**Figure 2:**
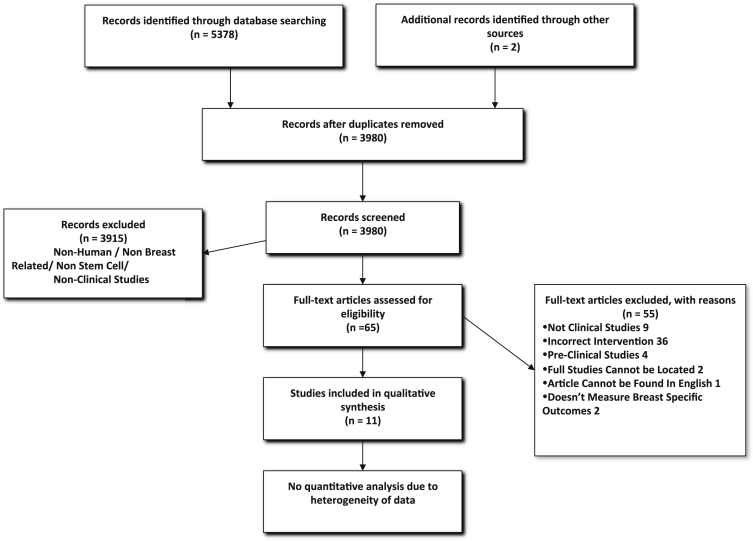
Search history following the PRISMA guidelines.

The literature and database searches identified 11 studies for inclusion (Figure 2 provides a detailed breakdown of the search). The search of PubMed and Ovid yielded 3553 and 1825 studies, respectively. Searches through the bibliographies of the identified articles and relevant publications resulted in the addition of two further studies. A number of incomplete trials were identified from searches on clinical trials registers that will be analysed separately in the discussion section of this paper.

### Overview of studies

The 11 studies included (Table 1) comprised of 336 participants with follow-up times that were both variable and occasionally inconsistent within the studies. They ranged from six months in Wang et al. 2015^19^ and Peltoniemi et al. 2013^20^ to 42 months in Yoshimura et al. 2008^12^ (a single patient was followed for this length of time). The majority of trials (n = 9) were observational, with eight being prospective and one retrospective with none being randomized. The further two studies comprised one case study^21^ and one case series.^12^ Six studies looked into CAL for breast augmentation. Five concerned the use of CAL in breast reconstruction to treat breast defects in patients undergoing cosmetic, congenital and reconstructive procedures.

### Patient demographic and operative details

All procedures carried out were single-stage procedures with no *in-vitro* expansion of the harvested stem cells. Studies described a similar surgical technique utilizing initial stab incisions, cannula insertion and subsequent injection of the fat graft into the breast in a multilayered and circular manner to optimize distribution. Weighted mean age and BMI of participants were 42.7 (SD 7.15) years and 21.87 (SD 1.89) kg/m^2^, respectively. Weighted mean fat harvested was 691.77 (Standard Deviation = SD 339.25) ml. Weighted mean fat injected into the left and right breast was 253.65 (SD = 20.05) and 265.51 (SD = 14.15) ml, respectively. When volume injected into each breast was not disclosed per breast this average was 174.97 (SD 63.69) ml/per breast. Fat was harvested from a number of anatomical locations with a number of different systems being used to process the fat as detailed in Table 2. Table 2 also summarizes data regarding cell count and viability within the fat grafts. A number of studies recruited women of solely Asian heritage (Table 1).^14^, ^23^, ^27^, ^29^, ^32^

### Quality assessment

**Figure 3:**
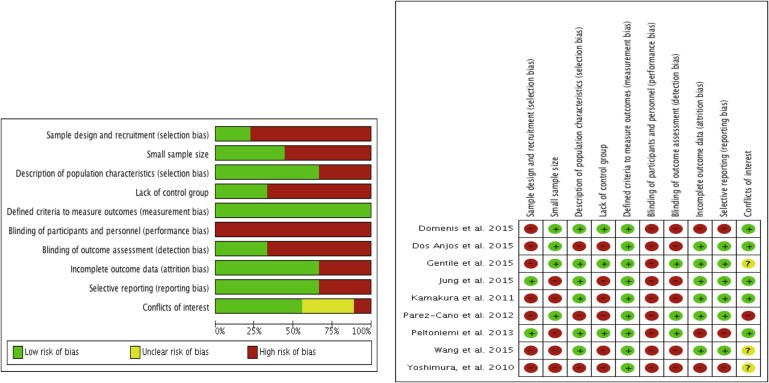
a/b – Risk of bias summary: review authors' judgements about each risk of bias item for observational studies.

Figure 3a/b summarizes the bias assessment that was carried out. A large number of the studies exhibited selection bias as they did not provide details regarding their recruitment process. Many studies did not report the demographic of their patient cohort in detail for an assessment of whether the results could be extrapolated to the general population. The criteria used to measure outcomes were pre-specified and contained mostly objective methods of assessment but did include more subjective assessments of patient/surgeon satisfaction. For example, in Kamakura et al. 2011,^22^ patients were asked to select a satisfaction rating from the options: excellent, good or fair with no focus on negative outcomes. Only three studies used comparison groups. In addition to this, one study Dos Anjos et al. 2015^23^ used a “sham control” where the comparison group received fat grafts that were enriched with a low level of adipose stem cells that showed no advantage over conventional autologous fat transfer. Three studies exhibited a high level of attrition bias: Yoshimura, et al. 2010,^24^ where 12 month data were collected for only six out of an original cohort of 15 patients and Peltoniemi et al. 2013,^20^ where two participants were excluded as their BMI had changed too much. In addition, when it was realized that the intervention showed no advantage over conventional autologous fat transfer the study was discontinued. In Domenis et al. 2015^25^ data was not collected for seven out of 30 patients. In a number of studies financial conflicts of interest were not declared with one study author declaring that he is an employee of Cytori Therapeutics (San Diego; USA): a regenerative-medicine company developing cell-based therapies from adult adipose tissue.

### Graft survival

A summary of graft survival outcomes and how they were measured in each study are presented in Table 3. Jung et al. 2015,^26^ Wang et al. 2015^19^ and Peltoniemi et al. 2013^20^ all reported negative outcomes in terms of graft volume. Jung et al. 2015^26^ described a 43% decrease in breast volume, a figure similar to that seen in conventional autologous fat grafting. Peltoniemi et al. 2013^20^ is one of three studies that includes a comparison group and found that graft retention in its intervention group was 50% compared to 54% in the control – although there was no statistical difference between the two. Two of the largest studies, Gentile et al. 2015^27^ (n = 50) and Dos Anjos et al. 2015^23^ (n = 74), reported positive and statistically significant results. Gentile et al. 2015^27^ found an average graft retention of 49.25% using CAL vs. 39% in its control group and represents the second study incorporating a control group. Dos Anjos et al. 2015^23^ reported an average graft retention of 75% when using grafts that were high in their stem cell content compared to 50% in the group of patients that received low stem cell enriched grafts in what the authors describe as a ‘sham control group’. Domenis et al.,^25^ the final controlled trial, found graft retention to be better in its cell enriched group also – however, this measurement was made though ultrasonography. In all other studies, authors report positive findings although no comparison group was present, and the quantitative data presented does not indicate any meaningful advantage over existing techniques.^27^ Methods of measuring graft volume retention (summarized in Table 3) varied between studies and the case study by Calabrese et al. 2009^21^ did not report any quantitative measurement of this outcome. The majority of studies used magnetic resonance imaging (MRI) due to its reliability and accuracy in measuring graft volume,^28^ however a few studies opted for physical measurements (e.g. breast circumference). Yoshimura, et al. 2010^24^ and Dos Anjos et al. also used 3D measurement techniques.

### Complications

The complications that occurred during the course of each study are presented in Table 4. The overall complication rate across studies was 37%, with the most common side effect being calcification – comprising 83% of all complications. Fibrosis, and consequent hardening of the breast, was seen in a single case by Yoshimura et al. 2008^24^ and an isolated case of Mondor's disease (a rare condition that leads to thrombophlebitis of the superficial veins of the breast and anterior chest wall^29^) was reported by Dos Anjos et al. 2015.^23^ Five serious adverse effects were reported either during or immediately post-procedure by Pérez-Cano et al. 2012^30^ – but only two of these were described. They comprised of subcutaneous bleeding, which was thought to be due to the post-operative use of anti-coagulants, and bony metastasis; secondary to natural disease progression.

### Patient reported outcomes

Validated PROMs were only used in a single study.^30^ The study, by Pérez-Cano et al. 2012, represents the most comprehensive assessment of PROMs and used a number of objective scales to assess patient satisfaction. This included *Clough's Classification System* for breast reconstruction, the *Late Effects Normal Tissues* (*LENT*)*-Subjective Objective Management Analysis* scoring system and a *Quality of Life* assessment. Overall, at 12 months, 58 out of 67 surgeons and 45 out of 67 patients were reportedly ‘satisfied’ with aesthetic outcomes, and 57 out of 67 surgeons and 50 out of 67 patients reported satisfaction with regards to the whole treatment process, when taking into account all of the assessment methods used. Quality of life scores remained constant throughout the follow-up period. Three studies did not report any measure of patient reported outcomes (See Table 1). Of the studies that did report patient reported outcomes, the methods for data collection were not stated. For example, in the study by Calabrese et al. 2009,^21^ the patient reportedly stated their outcome as ‘excellent’ with no accompanying explanation of how this was justified. This also occurred in the study by Kamakura et al. 2011,^22^ where 75% of patients were reportedly ‘satisfied’ with outcomes. Wang et al. 2015,^19^ reported a single ‘unsatisfied’ patient and Yoshimura et al. 2008^12^ reported that ‘most patients were satisfied with their enlarged and soft breasts with a natural contour’ – and the surgeons also described the results as ‘generally satisfactory and encouraging’. When patients were asked if they would undergo repeat procedures, Peltoniemi et al. 2013^20^ reported that one patient answered ‘no’ and a further two were ‘unsure’ but offered no further justification of these responses.

The outcome assessment conducted by Gentile et al. 2015^27^ was based on objective criteria examining five outcomes, including:

(1) Presence of asymmetry, deformity, irregularity, dyschromia, dysaesthesia, paraesthesia, and pain;
(2) Results of the superoexternal quadrant, inferoexternal quadrant, superointernal quadrant, and inferointernal quadrant;
(3) Resorption of fat in one or more regions;
(4) Time of stabilization of the transplanted fat; and
(5) Need for retreatment.

Patients provided a yes/no or positive/negative evaluation in addition to a percentage value. All patients reported satisfaction with texture, softness and contour. A detailed breakdown incorporating all the outcomes assessed above was not provided.

Generally, the provision of patient reported outcomes in the studies identified was poor. Future studies, therefore, should only use validated PROMs such as the BREAST-Q that are used appropriately and time-critically. This will generate more meaningful patient insights and facilitate a more consistent degree of comparability between studies.

For additional analysis please see Supplementary Material Document 2.

## Discussion

Due to the infancy of this technique in breast augmentation and reconstruction, the current level of evidence surrounding CAL makes it difficult to draw conclusions for its use in the clinical setting. The majority of studies included in this review are positive in relation to graft retention, complication rates and patient or surgeon reported outcomes. However, there is a lack of methodological rigour – the absence of control groups in eight out of the 11 studies makes it difficult to ascertain the efficacy and safety of CAL. In addition, one of the three studies^20^, ^25^ that made use of a control group found no statistical advantage in using CAL in contrast to the other two controlled studies^25^, ^27^ that did find a difference. Evidently, further work is needed to assess the full effect, if any, of CAL for this indication. Peltoniemi et al. 2013^20^ highlighted that the conventional autologous fat transfer procedure takes around 90–150 min less time in addition to being $3000 cheaper than CAL. Any benefits that CAL may bring need to be weighed against such drawbacks.

One of the key limitations that this systematic review highlights is the short follow-up times that are insufficient to assess the long-term implications of using CAL. This is of particular concern as pre-clinical studies have shown the use of stem cell enriched autologous fat to increase rates of local cancer occurrence^31^ and metastasis.^32^ We also found that calcification was the second most common complication of CAL, occurring in four out of 336 patients. Although in all cases this calcification was found not to be of malignant origin, the presence of calcification may lead to false positive results on mammography causing undue stress – this can be mitigated through use of expert radiologists who can readily distinguish malignancy from fat changes in the context of fat grafting.^33^

The field of plastic surgery is widely adopting the use of quality of life measures.^34^, ^35^ Although the majority of studies did acknowledge patient satisfaction – the methodology was generally not described and as such, their reliability is limited. Most notably, in Kamakura et al. 2011,^22^ patients were questioned about their satisfaction with their treatment outcomes in terms of three possible responses: excellent, good, or fair, with no option to report any potential negative outcomes – demonstrating a considerable element of bias. Therefore, the use of validated PROMs, such as BREAST-Q, should be routinely utilized. These validated PROMs are superior to conventional methods of collecting patient reported outcomes and are considered *reliable* (in that they measure how the procedure has influenced the patient and not any other factor), *valid* (in that they are objective in what they intend to measure) and *sensitive* (in their ability to detect change in a patient).^36^, ^37^ The use of such measures is therefore twofold: firstly, this would allow a consistent measure of satisfaction and well-being that could be compared between studies to collect relevant information while minimizing bias; secondly, BREAST-Q covers a broad range of domains including psychosocial, physical and sexual well being in addition to satisfaction with breasts, care and outcome and how this relates to the patients expectations.^38^ In doing so, reliable data could be pooled to take a more patient centred approach to improving CAL. It could also provide insight into patient understanding of the procedure including its limitations to foster improvement in the acquirement of informed consent.

**Figure 4:**
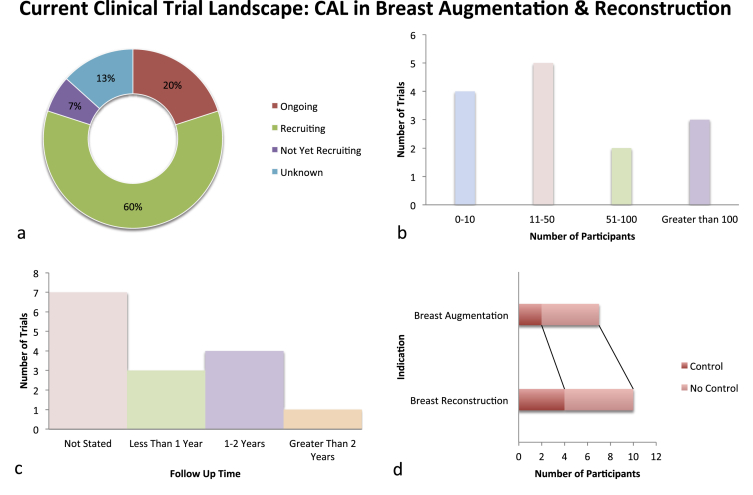
The majority of studies are still in the early stages of translation in an attempt to ascertain the efficacy and safety of the procedure (a) and a major proportion of the trials plan on recruiting a larger number of participants (Note sample number unknown for one study) (b). An increasing number of trials plan on employing comparison groups especially for breast reconstruction (Note that some trials are investigating CAL for both breast augmentation and reconstruction so these are shown separately in this figure) (d). On the contrary future trials still fail to show a follow-up time long enough to demonstrate long-term effects (c).

To assess the current clinical trial landscape, active clinical trials involving CAL in breast-related procedures were identified through searches of relevant clinical trial registries (see Figure 4). In total, 15 active studies were identified – the majority in early stages of translation (9 in phase two clinical trials). This is in line with what would be expected for an intervention in the early stages of development. In contrast to the studies previously mentioned, a significant proportion of the trials already have or are planning to recruit large numbers of participants, with the largest aiming for 440. Whether this is achievable is open for debate but may offer an explanation as to why such a significant proportion (60%) are still in the recruitment stage. Six out of the 15 future trials intend to use a comparison group, for more rigorous data analysis into the effects of CAL in breast reconstruction or augmentation. Our systematic review highlighted inadequate trial follow-up times and a failure to address long-term outcomes, particularly surrounding the risk of malignancy. This is a major concern for both patients and clinicians and will likely dictate the viability of the procedure and its potential for universal application in breast-related and other procedures.

## Limitations

Limitations of this study include the small sample of patients (n = 336) and the high levels of selection bias found within the studies. Only three of the studies included control groups, making it difficult to compare CAL to conventional autologous fat grafting. Follow-up times varied within studies and were too short to detect long-term outcomes of interest – notably the risk of malignancy. The studies also employed heterogeneous methods to measure graft survival with a corresponding lack of validity regarding the methods of assessing patient satisfaction levels post-operatively. In addition, a number of studies failed to declare financial conflicts of interest and in one study an author declared himself an employee a company developing cell-based therapies from adult adipose tissue. The large degree of heterogeneity meant that a meta-analysis to calculate the true effect size of cell assisted lipotransfer for the outcomes of interest discussed within this systematic review was not feasible.

## Conclusion

The literature indicates that CAL may be a promising surgical technique. Presently, studies demonstrate high levels of bias, lack control groups and display considerable heterogeneity, making the generalizability of study results and effect size unclear. Furthermore, lack of long-term follow-up data and associated concerns of malignant risk require mitigation. Further rigorous randomized controlled trials are needed to investigate the procedure's full clinical efficacy and safety profile that should also make use of validated PROM's.

## Financial disclosure

Zeeshaan Arshad: None.

Lindsey Karmen: None.

Rajan Choudhary: None.

James A. Smith, BSc: None.

Olivier A. Branford, PhD: None.

David A. Brindley, DPhil: This article represents the authors' individual opinions and may not necessarily represent the viewpoints of their employers. Brindley is a stockholder in Translation Ventures Ltd. (Charlbury, Oxfordshire, UK) and IP Asset Ventures Ltd. (Oxford, Oxfordshire, UK), companies that among other services provide cell therapy biomanufacturing, regulatory, and financial advice to pharmaceutical clients. Smith is a consultant with IP Asset Ventures Ltd. Brindley also is subject to the CFA Institute's codes, standards, and guidelines, so he must stress that this piece is provided for academic interest only and must not be construed in any way as an investment recommendation. Additionally, at time of publication, Brindley and the organizations with which he is affiliated may or may not have agreed and/or pending funding commitments from the organizations named herein.

David Pettitt, MBChB: None.

Ben Davies, DPhil: None.

## Figures and Tables

**Table 1:** An overview of the included studies discussing key aspects such as study design, details regarding follow-up, conclusions and the main limitations of each trial.

Article ID	Study design	Intervention	Country	Follow-up details	Main conclusions	Use of validated PROMS and patient reported outcomes
Calabrese et al. 2009	Single patient case study	Breast reconstruction	Italy	Aesthetic result measured at 10 months; graft retention at 3 months and 17 month oncological follow-up	Positive outcome/sufficient and reliable way to restore breast volume with natural shape/no post-operative complications/patient evaluated results as excellent	No use of validated PROMS. No explanation of how information regarding patient satisfaction was gathered.
Kamakura et al. 2011	20 Japanese women in prospective, non-randomized open label study	Breast augmentation	Japan	9 months with check up at 2 weeks and then 1, 3.6 and 9 months	Positive outcome/improvement in baseline breast volume/method is safe as no serious or adverse side effects/physician and patient satisfaction at 69% and 75% respectively/cysts in two patients	No use of validated PROMS. Patients were asked their overall satisfaction with treatment results in terms of three possible responses: excellent, good, or fair
Gentile et al. 2015	50 patients (10 control) in non-randomized control trial	Breast reconstruction	Italy	2, 7, 15, 21, and 36 weeks and then annually	Positive outcome/statistically significant improvement in breast contour and 3D volume/ultrasound showed cysts in 45.83% of patients/cytosteatonecrotic areas were observed with ultrasound in 12.5%/no serious complications	No use of validated PROMS – although there was use of objective criteria to measure patient satisfaction.
Wang et al. 2015	12 Chinese women in open labelled non-randomized study	Breast augmentation	China	6 month follow-up with one check up at 3 months	Mixed conclusion/the resorption of grafted fat at the 6 months post-operatively was 51.84% that presents no statistical advantage over existing techniques/cysts and nodules were detected in 2 cases/no calcification was found. Only 1 patient was unsatisfied with cosmetic outcome. Study displayed a satisfactory augmented volume with little complications using CAL for breast augmentation	No use of validated PROMS. No explanation of how information regarding patient satisfaction was gathered.
Yoshimura et al. 2008	Case series of 40 Japanese patients	Breast augmentation	Japan	19 of the patients have been followed for longer than 6 months/longest follow-up has been 42 months	Procedure is effective and safe/positive cosmetic outcome/cysts in two patients/two patients experienced fibrosis of breast and sternum/created a more natural breast contour but lower height than implant augmentation/all patients were described as satisfied	No use of validated PROMS. No explanation of how information regarding patient satisfaction was gathered.
Jung et al. 2015	Prospective study of 5 women	Breast augmentation	Korea	Follow-up at 3 months and 1 year; will continue to 2 years every 6 months	Negative as no apparent benefit to addition of SVF/oil cysts in 3 of the 10 breast/1 patients had nodule/only about half of grafted volume was present at 1 year/outcome better in nulliparous women	No report of patient reported outcomes.
Yoshimura, et al. 2010	Prospective study of 15 Japanese women	Breast augmentation	Japan	8 patients followed for more than 12 months with maximum follow-up of 18 months	Positive/clinical results were satisfactory/no major abnormalities were seen on magnetic resonance imaging or mammogram after 12 months/all patients report satisfaction	No use of validated PROMS. No explanation of how information regarding patient satisfaction was gathered.
Dos Anjos et al. 2015	Retrospective, non-randomized trial including 74 women	Breast reconstruction	Spain	7 days–540 days post-operatively	Final volume retention in the long-term was higher with high cell-enhanced fat grafts. Complications include Mondor's disease/9 cases of subcutaneous benign lumps/oil cysts in 14 patients/no intraoperative complications.	No report of patient reported outcomes.
Peltoniemi et al. 2013	Prospective controlled study with 18 women (8 controls)	Breast augmentation	Finland	6 months	Negative/no additional benefit to enrichment of graft SVF so not worth increased cost and risk. 1 patient in both control and intervention group developed small cysts.	No use of validated PROMS. No explanation of how information regarding patient satisfaction was gathered.
Pérez-Cano et al. 2012	Prospective trial with 71 patients	Breast reconstruction	Europe	12 months with 6 month check up	Positive/improvement of breast contour in 54 cases/no serious adverse events/no reported local cancer recurrences/injection site cysts in ten patients/50 patients and 57 investigators reported satisfaction/subcutaneous bleeding following liposuction and pelvic bone metastasis	Only study to use validated PROMs. Used a number of objective and subjective scales to assess patient satisfaction including Clough's classification system for breast reconstruction, Late Effects Normal Tissues (LENT)-Subjective Objective Management Analysis scoring system and a Quality of Life assessment.
Domenis et al. 2015	30 patients	Breast reconstruction	Italy	12 months with a 6 month check up	Patients treated with SVF enhanced fat grafts demonstrated superior outcomes/no mention of complication	No report of patient reported outcomes.

**Table 2:** This table provides a summary of operative details for each included study.

Article ID	Number of participants	Mean age (years)	Mean BMI (kg/m^2^)	SVF isolation method	Cell count in graft	Mean cell viability (%)	Mean volume harvested (ml)	Volume injected (ml) (left breast/right breast)	Site of fat harvest
Calabrese et al. 2009	1	37	–	Celution system	–	–	355	–	Abdomen and external thighs
Kamakura et al. 2011	20	35.6 (range 21–52)	–	Celution system	3.42 × 10^5^ ± 1.39 × 10^5^/g	85.3 ± 6.2	1026.5 (range, 660–1125)	235.1 ml (range 166–290)/244.9 ml (range 166–330)	Thighs, hips, buttocks, or abdominal area
Gentile et al. 2015	50	Range 19–60	–	Celution, Lipokit Medikhan, Fatstem, and Mystem systems	–	98	–	93.54 (range 50–150)	–
Wang et al. 2015	12	32 (range 28–56)	22.10	Lipokit Medikhan system	Nucleated cell numbers 2.74 ± 1.07 × 10^7^/g	–	750	256 (range 198–330)	Thighs, flanks, and/or lower abdomen
Yoshimura et al. 2008	40	35.8 (range 20–62)	19.1 ± 1.9	–	–	–	1111.8 ± 164.0	268.1 ± 47.6/277.3 ± 39.1	Thighs, abdomen and lower legs
Jung et al. 2015	5	34.4 ± 9.15	20.18 ± 2.84	–	34.76 × 10^6^ ± 27.14 × 10^6^ SVF cell count	–	574.4 ± 152.58	196.2 ± 49.69/246.2 ± 62.99	–
Yoshimura, et al. 2010	15	37.1 ± 12.5	19.5 ± 1.4	–	–	–	–	259 ± 39/268 ± 29	–
Dos Anjos et al. 2015	74	38.58	21.58	GID SVF-1 device	5.83 × 10^5^ ± 2.88 × 10^5^/ml	82.79 ± 8.14		249.9	Infraumbilical area and flanks
Peltoniemi et al. 2013	18	51 (range 29–58)	23.4 (range 20.3–32.5)	Celution system	–	–	–	177 (range 122–298)/180 (range 122–258)	
Pérez-Cano et al. 2012	71	52 (37–68)	24.5 (range 17–31)	Celution system	2.95 × 10^5^ stromal vascular cells per ml	86.6	364 (range 223–570)	140 (range 35–250)	
Domenis et al. 2015	30	48 (19–74)	21.4 (19.8–32.8)	Celution, Lipokit Medikhan, Fastem Corios system	–	–	–	–	Abdomen, hips, and trochanteric area

**Table 3:** A summary of the graft volume retention and how it was measured.

Article ID	Number of participants	Breast/graft volume measurement method	Result
Calabrese et al. 2009	1	Graft retention in mastectomy flap evaluated with MRI and ultrasound at 3 months.	Patient evaluation as excellent, surgeon as good
Kamakura et al. 2011	20	Measured change in breast size (BRM) – circumferential breast (B) and circumferential chest (C) were measured and subtracted through physical examination to calculate this.	BRM on average increased by 3.3 cm
Gentile et al. 2015	50 patients (10 control)	3 Methods used: team evaluation, MRI and USS, patient self evaluation.	49.25% of volume maintained on average in intervention group vs. 39% in control
Wang et al. 2015	12	MRI, 3T whole body scanner, 3 radiologists.	Resorption rate at 6 months from 19.99% to 71.22%, mean 51.84%
Yoshimura et al. 2008	40	Breast circumference (chest circumference at the nipple minus the chest circumference at the inframammary fold) was measured through physical examination.	Circumference increase 100–200 ml after injection of 270 ml
Jung et al. 2015	5	Breast volume determined by volume rendering technique, using MRI. Each breast outlined anteromedially along inner surface of skin and posteriorly along the anterior surface of pectoral muscle.	One year after CAL, breast had decreased to 47% of initial post-operative volume
Yoshimura, et al. 2010	15	Mammography, MRI, photography, videography and 3D measurements, to allow for volumetric evaluation of breast mound.	Right mean 155 ± 50 ml Left mean 143 ± 80 ml At 12 months Graft take ranged from 40 to 80%
Dos Anjos et al. 2015	74	3D imaging scan utilized to quantify volume changes. ARTEC MHT 3D scanner, superimposed to measure difference. Scans Pre and post LD volume.	75% and 50% breast volume retention in high and low SVF cell enrichment groups, respectively
Peltoniemi et al. 2013	18 women (8 controls)	MRI scans before and 6 moths after surgery to exclude complications. Volume analysis performed by a blinded independent examiner.	54% graft survival in control group and 50% in intervention group
Pérez-Cano et al. 2012	71	T1 weighted MRI at baseline, 6 and 12 months post index treatment. Scored by 2 independent radiologists blinded to patient sequence using 5 point Likert Scale for breast defect and contour.	57 of 67 patients reported satisfaction. Independent radiographic core laboratory assessment reported improvement in the breast contour of 54 out of 65 patients based on blinded assessment of MRI sequence
Domenis et al. 2015	30 (16 control)	USS measurement of subcutaneous thickness in reconstructed breast acquired preoperatively, 6–12 months post op.	Patients treated with SVF enhanced grafts demonstrated at 6 months a significant superior gain of thickness of both central and superior medial quadrants with respect to patients treated with standard lipotransfer

**Table 4:** Complications that occurred during the course of each study. It should be noted that each complication was considered to be an independent event as information regarding multiple complications in single patients was not available. Calcification is by far the most commonly cited complication using CAL. Note that no information regarding complications was reported in Domenis et al. 2015.

Article ID	Number of participants	Cysts	Calcification	Cancer occurrence	Operative complications	Other complications
Calabrese et al. 2009	1	None	None	None	None	None
Kamakura et al. 2011	20	2	2	None	None	Fat necrosis
Gentile et al. 2015	50 (10 control)	23	None	None	None	Cytosteatonecrotic areas in 6 patients
Wang et al. 2015	12	2	None	None	None	Swelling and ecchymosis for one month/2 nodules
Yoshimura et al. 2008	40	2	2	None	None	Fibrosis of sternum and breast tissue
Jung et al. 2015	5	3	None	None	None	1 nodule
Yoshimura, et al. 2010	15	None	None	None	None	None
Dos Anjos et al. 2015	74	14	None	None	None	Mondor's disease in one patient
Peltoniemi et al. 2013	18 (8 controls)	1	None	None	–	None
Pérez-Cano et al. 2012	71	10 (46 sub-clinical)	None	Yes (pelvic bone metastasis)	Five serious adverse events including subcutaneous bleeding following liposuction.	None
