# Supplementary material for: Cell assisted lipotransfer in breast augmentation and reconstruction: A systematic review of safety, efficacy, use of patient reported outcomes and study quality
Source: JPRAS Open. 2016 Aug 24;10:5–20. doi: 10.1016/j.jpra.2016.08.004 (PMC5193151; doi:10.1016/j.jpra.2016.08.004)
Supplement: Supplementary file 1 [file mmc1.docx]

Supplementary Material Document 1 – Methods

A systematic review was carried out according to the PRISMA guidelines with pre-specified methods of analysis and inclusion/ exclusion criteria. The search terms used can be found at the end of this document. A search was carried out on PubMed, the Ovid database, International Standard Randomised Controlled Trial Number (ISRCTN), clinicaltrials.com, EU Clinical Trials Registers and the NIPH Clinical Trials Search. Searches were carried out for articles published between 2007/01/01 and 2015/12/31 (as 2007 was the year of the first human clinical study). Bibliographies of identified studies and other relevant articles, including recent review articles, were searched manually for any publications the searches may have overlooked. Authors of non-English articles were contacted to obtain translated versions and the authors of conference and/or meeting abstracts were contacted for details regarding results.

**Eligibility Criteria and Study Selection**

Two independent reviewers independently searched through all identified abstracts to select studies that met the specified criteria. Any discrepancies were resolved via discussion. The population and intervention included any female patient who underwent breast augmentation or reconstructive surgery utilizing adipose derived stem cells. It should be noted that no report of this procedure being used on a male subject was found. The primary outcome of interest was a measure of graft survival (e.g. as determined by change in breast circumference measurements). The rationale for selecting this outcome measure was twofold; i) it provides a quantitative representation of procedural efficacy and ii) provides a means of comparing CAL to existing methods. Secondary outcome measures included complication rates with a particular focus on intraoperative complications as well as cyst formation, calcification and cancer occurrence. Further data was sought concerning the use of fully validated PROMS (patient reported outcome measurements) as well as information pertaining to study quality such as recruitment, follow up and reporting of demographic data. All studies involving humans were included (including single case reports) regardless of whether or not they included a control group due to the limited number of clinical studies in the area. Non-English studies were included if the full article could be obtained in English. Human studies that reported the use of CAL in breast augmentation/ reconstructive surgery and other indications were included if the studies reported outcomes specifically for the breast related procedures.

**Data Collection and Assessment of Quality and Bias**

A data extraction sheet was developed to retrieve the relevant data from each article. Data extraction was completed by two independent reviewers, and any discrepancies were resolved via discussion. Data extracted included methods of recruitment and participant selection, including inclusion and exclusion criteria that may have led to bias in the results. Information regarding the characteristics of the patient population (age, BMI, patient problem) in addition to details of the procedure itself, pre-operative assessments, postoperative management, details of follow-up and methods of data collection in relation to the outcomes of interest was sought. Numerical data that was collected included any measurement of graft survival, information regarding patient reported outcomes and complication rates.

A critical appraisal of each article was carried out using the guidelines for evaluating observational and case studies on the Equator Network website (http://www.equator-network.org). An objective assessment was made of bias present in the studies that were observational in nature using Review Manager (RevMan), version 5.3 (The Nordic Cochrane Centre, The Cochrane Collaboration, Copenhagen, The Netherlands). This was carried out by two independent reviewers with any discrepancies resolved through discussion.

**Summary Measures and Data Analysis**

Graft volume retention was the primary outcome measure. In addition to this, data regarding complication rates and cosmetic outcomes was extracted. Data were too heterogeneous for meta-analysis and as such data is presented in a descriptive format according to the PRISMA guidelines. RevMan software was used to objectively assess and present information regarding bias under the following headings: selection, measurement, performance, detection, attrition and reporting bias in addition to the presence of any financial conflicts of interest, presence of control groups and sample size.

Search Terms

The boxes below show the search terms that were used for each search engine and clinical trial registry.

PubMed

("cells"[MeSH Terms] OR "cells"[All Fields] OR "cell"[All Fields]) AND assisted[All Fields] AND lipotransfer[All Fields]) OR (Progenitor Enriched[All Fields] AND ("adipose tissue"[MeSH Terms] OR ("adipose"[All Fields] AND "tissue"[All Fields]) OR "adipose tissue"[All Fields])) OR (Cell Enriched[All Fields] AND Fat[All Fields] AND ("transplantation"[Subheading] OR "transplantation"[All Fields] OR "grafting"[All Fields] OR "transplantation"[MeSH Terms] OR "grafting"[All Fields])) OR (stromal[All Fields] AND ("blood vessels"[MeSH Terms] OR ("blood"[All Fields] AND "vessels"[All Fields]) OR "blood vessels"[All Fields] OR "vascular"[All Fields]) AND fraction[All Fields]) AND ("2007/01/01"[PDAT] : "2015/12/15"[PDAT])

OR

("Breast Surgery"[All Fields] OR "Adipocytes/transplantation*"[Mesh]) OR ("Transplantation, Autologous/adverse effects"[Mesh] OR "Transplantation, Autologous/methods"[Mesh]) AND (Fat[All Fields] AND ("injections"[MeSH Terms] OR "injections"[All Fields] OR "injection"[All Fields])) AND ("2007/01/01"[PDAT] : "2015/12/15"[PDAT])

OR

("Adipocytes/transplantation*"[Mesh] OR ("Adipocytes/cytology"[Mesh] OR "Adipocytes/surgery"[Mesh])) OR ("Adipose Tissue/cytology"[Mesh] OR "Adipose Tissue/surgery"[Mesh] OR "Adipose Tissue/transplantation"[Mesh]) OR ("Stromal Cells/cytology"[Mesh] OR "Stromal Cells/surgery"[Mesh]) OR "Stromal Cells/transplantation"[Mesh] OR "Mesenchymal Stem Cell Transplantation/methods"[Mesh] AND "Mammaplasty/methods"[Mesh] OR "Breast Implantation/methods*"[Mesh] OR "Lipectomy/methods"[Mesh] OR "Breast Implants/adverse effects*"[Mesh] OR (("Adipocytes/cytology"[Mesh] OR "Adipocytes/surgery"[Mesh]) OR ("Adipose Tissue/cytology"[Mesh] OR "Adipose Tissue/surgery"[Mesh] OR "Adipose Tissue/transplantation"[Mesh]) OR ("Stromal Cells/cytology"[Mesh] OR "Stromal Cells/surgery"[Mesh]) OR "Stromal Cells/transplantation"[Mesh] OR "Mesenchymal Stem Cell Transplantation/methods"[Mesh] AND "Breast Surgery"[All Fields] OR "Adipocytes/transplantation*"[Mesh]) OR ("Transplantation, Autologous/adverse effects"[Mesh] OR "Transplantation, Autologous/methods"[Mesh]) AND ("2007/01/01"[PDAT] : "2015/12/15"[PDAT])

ClinicalTrials.Gov

(‘Adipocyte’ OR ‘Adipose’ OR ‘Stromal’ OR ‘Mesenchymal Stem’ OR ‘Mesenchymal stromal cells’ OR ‘Adipose-Derived Regenerative’ OR ‘Cell assisted lipotransfer’ OR ‘Stromal vascular fraction’ OR ‘Cell Enriched Fat Grafting’ OR ‘lipofilling’) AND 'Breast'

EU Trials Register

Adipose/ Mesenchymal Stem/ Cell Enriched Fat Grafting/ Fat Injection(s)

NIPH Clinical Trials Search

Adipose/ Stromal/ Mesenchymal Stem/ Cell-assisted lipotransfer/ Fat Injection

ISRCTN

- Adipocyte/ Adipose/ Stromal/ Mesenchymal Stem/ Mesenchymal stromal cells/ Adipose-Derived Regenerative/ Cell assisted lipotransfer/ Stromal vascular fraction/ Cell Enriched Fat Grafting/ Mammaplasty/ Breast Implantation OR Implants/ Autologous Transplantation/ Free fat transfer/ Adipose-Derived Regenerative /Lipofilling

Ovid

1. Adipocytes/cy, tr [Cytology/ Transplantation]
2. Adipose Tissue/cy, cu, tr [Cytology/ Surgery/ Transplantation]
3. Stromal Cells/cy, tr [Cytology/ Transplantation]
4. Mesenchymal Stem Cell Transplantation/mt [Mountains]
5. 1 or 2 or 3 or 4
6. Mammaplasty/mt [Methods]
7. Breast Implantation/ or “Prosthesis and Implants”/
8. Lipectomy/mt [Methods]
9. Breast Implants/ae [Adverse Effects]
10. 6 or 7 or 8 or 9
11. 5 and 10
12. Breast surgery.mp.
13. Adipocytes/ tr [Transplantation]
14. Transplantation, Autologlous/ae, mt [Adverse Effects/ Methods]
15. 12 or 13 or 14
16. 5 and 15
17. 11 or 16
18. Cell assisted lipotransfer.mp.
19. Stromal vascular fraction.mp.
20. Proginator enriched adipose.mp.
21. Fat grafting.mp.
22. 18 or 19 or 20 or 21
23. 17 or 22
