# Supplementary material for: Cell assisted lipotransfer in breast augmentation and reconstruction: A systematic review of safety, efficacy, use of patient reported outcomes and study quality
Source: JPRAS Open. 2016 Aug 24;10:5–20. doi: 10.1016/j.jpra.2016.08.004 (PMC5193151; doi:10.1016/j.jpra.2016.08.004)
Supplement: Supplementary file 2 [file mmc2.docx]

Supplementary Material Document 2 – Additional Analysis

**Recruitment of Patients**

Four studies did not specify any inclusion or exclusion criteria^1,2,3^ including the retrospective study^4^ that did not state how patients were selected for analysis. In the other studies, the exclusion criteria were predominantly for safety purposes with common criteria including contradictions to surgery such as smoking, pregnancy or cardiovascular disease. In some instances, a specified time period had to have lapsed from any form of malignancy, such as in Jung et al 2015^5^ that objectively screened potential patients using the BI-RADS scale. This scale uses imaging techniques to allocate a level of risk to breast lesions^6^. These criteria were created to maintain the safety of all participants and would have been appropriate to apply to women having the procedure in the general population. The study^1^ included all healthy women that had undergone breast augmentation for cosmetic purposes and represents the most widely inclusive study identified in this systematic review. Some studies, notably Kamakura et al. 2011^7^, excluded patients with thin or flaccid skin. Peltoniemi et al. 2013^8^ only included women with symmetrical breasts resulting in potential bias and favorable cosmetic outcomes – interestingly, they still concluded that CAL had no additional benefits over conventional autologous fat transfer.

**Follow-Up**

Follow-up was predominantly of a short duration with the longest being 42 months for a single patient in a cohort of 40 individuals (Yoshiumura et al. 2008). The shortest follow-up time was in the study by Peltoniemi et al. 2013^8^ and Wang et al. 2015^2^ where follow-up times lasted just six months in each case. In both of these studies there were considerable gaps in the follow-up period that could have resulted in some self-limiting complications, such as cyst formation or discomfort, being missed. Some studies such as Yoshiumura et al. 2008^1^ followed a proportion of patients for longer than others – the reasons for this or how these patients were selected is not specified. Follow-up times are insufficient to determine the long-term effects of CAL including the potential risk of malignancy. Some studies however did plan to follow-up patients annually or biannually via in-person consultations or other remote means of communication such as email or telephone. The assessments they intended to perform during follow-up were not specified.

Bibliography

1. Yoshimura K, Asano Y, Aoi N, et al. Progenitor-enriched adipose tissue transplantation as rescue for breast implant complications. *Breast J* 2010;16:169-175

2. Wang L, Luo X, Lu Y et al. Is the Resorption of Grafted Fat Reduced in Cell-Assisted Lipotransfer for Breast Augmentation? *Ann Plast Surg* 2015;75;128-134

3. Domenis R, Lazzaro L, Calabrese S, et al. Adipose tissue derived stem cells: in vitro and in vivo analysis of a standard and three commercially available cell-assisted lipotransfer techniques. *Stem Cell Res Ther* 2015;6;2

4. Dos Anjos S, Matas-Palau A, Mercader J et al. Reproducible Volume Restoration and Efficient Long-term Volume Retention after Point-of-care Standardized Cell-enhanced Fat Grafting in Breast Surgery: *Plast Reconstr Surg - Glob Open* 2015;3;e547

5. Jung HK, Kim CH, Song SY. Prospective 1-Year Follow-Up Study of Breast Augmentation by Cell-Assisted Lipotransfer. *Aesthetic Surg J Am Soc Aesthetic Plast Surg* 2015

6. Koning JL, Davenport KP, Poole PS et al. Breast Imaging-Reporting and Data System (BI-RADS) classification in 51 excised palpable pediatric breast masses. *J Pediatr Surg* 2015;50;1746-1750.

7. Kamakura T, Ito K. Autologous cell-enriched fat grafting for breast augmentation. *Aesthetic Plast Surg* 2011;35;1022-1030

8. Peltoniemi HH, Salmi A, Miettinen S, et al. Stem cell enrichment does not warrant a higher graft survival in lipofilling of the breast: a prospective comparative study. *J Plast Reconstr Aesthetic Surg JPRAS* 2013;66;1494-1503
